# Long-term use of an assistive exercise product in young people with cerebral palsy who are non-ambulant is a balancing act—a phenomenographic study

**DOI:** 10.3389/fresc.2026.1927259

**Published:** 2026-09-23

**Authors:** Jenny Gårdling, Katarina Lauruschkus, Åsa B. Tornberg

**Affiliations:** 1 Department of Health Sciences, Lund University, Lund, Sweden; 2 Department of Nursing and Integrated Health, Kristianstad University, Kristianstad, Sweden

**Keywords:** assistive exercise product, caregivers, cerebral palsy, family caregivers, phenomenography, professional caregivers, young people

## Abstract

**Introduction:**

According to the World Health Organisation (WHO), children should be physically active, including exercise, for 60 min a day, and limit sedentary behaviour across the week regardless of physical disabilities. Children with cerebral palsy who are non-ambulant (CP-NA), with Gross Motor Function Classification System Expanded & Revised IV to V, are known to be sedentary up to 92% of the time they spend awake and are dependent on assistive technology and assistive exercise products (AEP), such as the Innowalk. The aim of this study is therefore to explore the family and professional caregivers’ perceptions of the long-term use of the AEP after three to five years of use.

**Method:**

An inductive phenomenographic design was used, with the aim of describing and understanding variations in how individuals perceive a phenomenon; i.e., what something is and how something is perceived. 11 family caregivers and one professional caregiver to 11 young people (11–24 years) with CP-NA were interviewed individually using a semi-structured interview guide, after having used the AEP for three to five years. The analysis of the interviews followed the iterative steps of phenomenographic design.

**Results:**

Long-term use of the AEP was described by caregivers as a life-long project involving balancing between maintaining control and aspects that were beyond their control in everyday life to provide physical activity and exercise in the AEP. When the caregiver managed to reach such a balance, and were able to provide physical activity and exercise in the AEP, it was experienced as rewarding, creating a feeling of meaningfulness.

**Conclusion:**

Long-term use of the AEP requires substantial caregiver coordination and support from surrounding services. When such support is available, the AEP may contribute to meaningfulness and improved quality of life for both the young person and their caregivers.

## 1 Introduction

According to the World Health Organisation (WHO), children and adolescents aged 5–17 years old are recommended to perform at least an average of 60 min per day of moderate to vigorous physical activity, including exercise, and limit sedentary behaviour across the week, regardless physical disabilities (1).

Globally, 19% of children (11–17 years) meet WHO's recommendation of 60 min physical activity per day (1) at a moderate intensity and only 15% of children in Sweden meet WHO's recommendations (2). This warrants action to increase physical activity among children, since habits such as physical activity are set early in life (3) and facilitate physical activity levels throughout childhood into adulthood.

Daily physical activity is even lower for children with physical disabilities, who are often excluded from public arenas where physical activity and exercises are performed (4). They are excluded because of their disability, since physical activity facilities are usually not adapted to their needs. This lack of adaptation can cover multiple aspects; from not being able to enter the facility through a door that is not wide enough, to not being able to use the physical activity equipment at the physical activity centre (5). According to the United Nations’ (UN) Agenda 2030, children with disabilities have the same rights to good health and healthcare through physical activity as other children. All children have the right to participate in physical activity and exercise, and those who are unable to engage independently have the right to adequate/sufficient support (6, 7).

Physical activity is defined as any physical movement that is produced by skeletal muscles and requires energy expenditure (8). While ‘physical activity’ refers to any bodily movement, exercise is a subcategory of physical activity and refers to bodily movement that is planned, structured, and repetitive, with the purpose of improving or maintaining physical fitness and health. To enable physical activity and exercise among children with disabilities, specialised assistive technology is needed (9).

Cerebral Palsy (CP) is the most common physical disability in childhood. Approximately 1.6/1,000 children have CP with affected muscle tone, movement, and motor skills, often accompanied by pain, epilepsy, and intellectual, communicational, and behavioural impairments (10).

The degree of motor impairment in CP is extremely variable and classified with the 5-level Gross Motor Function Classification System Expanded & Revised (GMFCS-E&R) (11). Children with CP who are non-ambulant (CP-NA) (GMFCS-E&R levels IV and V) cannot stand or walk independently, and earlier studies have shown low physical activity levels in this group. Children with CP-A (GMFCS-E&R levels I–III) can either walk and/or stand independently or with a walking aid.

Assistive technology is defined according to WHO as the application of organised knowledge and skills related to assistive products, including systems and services (9). An assistive product is a product specially produced to promote well-being (9).

The assistive product, Innowalk (Madeformovement, Norway), is an assistive exercise product (AEP) that facilitates dynamic standing by enabling walking movements in an upright, weight-bearing position, similar to a motorised cross-trainer. Previous studies have compared static and dynamic standing, as well as high-intensity interval training and moderate-intensity continuous training performed in dynamic standing using the AEP Innowalk. Results from these studies have shown this AEP to give short-term effects such as increased energy expenditure (12), decreased spasticity (13), decreased medical use (14), and increased quality of life (14). In a hermeneutic phenomenological study when the AEP was introduced, interviewed family caregivers expressed that the AEP gave them hope in a challenging life situation in which their child was dependent on others to be physically active. The family caregivers were aware of the health benefits, but they were struggling with getting the family-time to fit (15).

To the best of our knowledge, there are no studies on the perceptions of long-term use of the AEP Innowalk. The purpose of this study is therefore to explore the family and professional caregivers' perceptions of the long-term use of the AEP after three to five years of use.

## 2 Method

### 2.1 Design

Phenomenography was used in this study, which is a descriptive, empirical, and content-oriented methodology (16). The aim is to describe and understand variations in how individuals perceive a phenomenon; i.e., what something is and how something is perceived. The outcome of the analysis, presented in the form of description categories, presents an attempt to describe and understand the variations of the perceived phenomenon. These categories are logically related, and together they constitute the outcome space (17). This manuscript is reported according to Consolidated criteria for reporting qualitative research (COREQ) checklist (18) (Supplementary 1).

### 2.2 Data collection

Participants were recruited from the original study cohort of a long-term follow-up of the two studies, ALTER (Dnr ISRCTN10569363) at five years' follow-up; and LEER (ClinicalTrail Dnr LU2019LEER), at three years' follow-up, comprising 33 children and young people with CP-NA. In both studies, the interventions were carried out in the participants’ home environment. The ALTER study compared four months of static standing with four months of dynamic standing exercises in the AEP Innowalk (Figure 1). In the LEER study, participants engaged in dynamic standing exercises, either through high-intensity interval training or moderate-intensity continuous training in the AEP Innowalk (Figure 1). All eligible families were invited to participate in the interviews. Of those caregivers whose child had previously participated in one of the research group's earlier studies and were familiar with the intervention, 12 participants, 11 family caregivers and one professional caregiver, including two family caregivers from the same family, agreed to participate. Family caregivers of 15 young people did not respond, three declined participation due to time constraints, three declined due to their child's poor health, and two of the young people had died since participating in the study. The participants were family caregivers and one professional caregiver; from hereon we will refer to them as caregivers. All with experience supporting young people with CP-NA during exercise using the AEP Innowalk. The young people were in median 17 [13:20] years at the follow-up, five with GMFCS-E&R level IV and six with GMFCS-E&R level V (Table 1). Participants had had prior contact with the authors KL and ÅBT through earlier studies.

**Figure 1:**
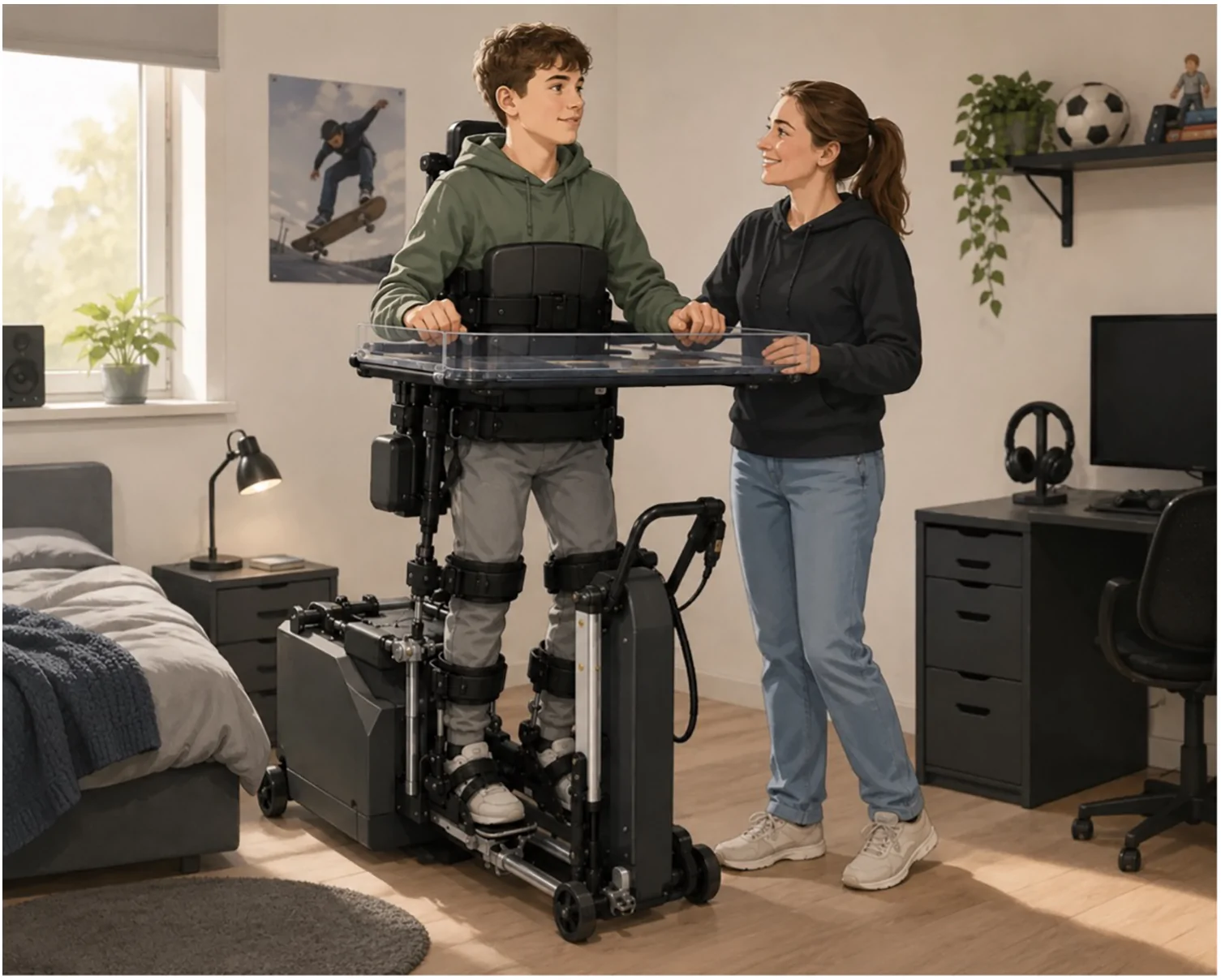
A young person exercising in the assistive exercise product (AEP) with the support of a caregiver.

**Table 1:** Participant characteristics.

Young people with CP with long-term experience of exercise in the Innowalk (*n* = 11)		Interview participants (*n* = 12)	
		Family caregivers	Professional caregiver
Male (*N*)	7	3	
Female (*N*)	4	8	1
Age (years)	11–24		
GMFCS-E&R level IV (*N*)	5		
GMFCS-E&R level V (*N*)	6		

CP, Cerebral Palsy; GMFCS-E&R, Gross Motor Function Classification System Expanded & Revised.

All participants received written and verbal information about the study prior to participation and provided informed consent. Participation was voluntary, and confidentiality was ensured. Ethical approval was obtained from the Swedish Ethical Review Authority (Dnr 2017/67, 2023-05139-02 and 2019-00106).

The interviews were performed from 2024 to 2025. Data were collected through semi-structured interviews exploring participants' experiences of long-term use of the AEP Innowalk. An interview guide with open-ended questions was developed by the research team based on the study aim and previous research. A pilot interview was conducted to verify the interview guide. As no changes to the interview guide was required the interview was included in the analysis (Supplementary 2). One author (KL), who had experience in qualitative interviewing and prior involvement in the research project, conducted all interviews according to participants' preferences, either at the participants' home (*n* = 3), by telephone (*n* = 6), or Zoom (*n* = 2). Although video was used during the Zoom interviews, only audio was recorded and transcribed. The pre-existing relationship may have facilitated trust and richer narratives, but may also have encouraged participants to emphasise positive experiences. The interviews lasted between 29 and 55 min and were audio-recorded and transcribed verbatim.

### 2.3 Data analysis

Data were analysed using a phenomenographic approach, aiming to describe variation in participants' perceptions of the long-term use of the AEP Innowalk. The analysis followed the iterative steps described by Dahlgren and Fallsberg (16) and further developed by Marton (17).

All transcribed interviews were read repeatedly by the authors KL, JG, and ÅBT to achieve familiarity with the data. The analysis was conducted collaboratively by the research team and involved several iterative steps. First, significant statements relevant to the study's aim were identified. These statements were then compared and grouped based on similarities and differences in how the phenomenon was described. Next, preliminary descriptions of the essence of each group were articulated. The process of grouping and articulating was repeated several times until consensus was reached.

The final description categories were developed to capture the qualitatively different ways in which participants experienced long-term use of the AEP Innowalk. These categories were continuously refined through discussions within the research team, ensuring that each description category was internally consistent and distinctly different from the others. Finally, the description categories were organised into an outcome space representing the logical relationships between them and illustrating the variation in participants' perceptions (Table 2).

**Table 2:** An example of the analysis procedure.

‘What’ they talk about	‘How’ they talk about the ‘what’ (perceptions)	Description categories
Careful planning Coordination Implementation	Everyday life relies on stability	Lifelong project leadership
Ensure continuity Sense of security Well-being affects daily life Creative solutions No room for spontaneity	Balance is important Structure is needed	Maintaining control in everyday life
Sustain existing level of physical activity Competent assistance Technical support Forced to find finances Entering adulthood	A struggle Rely on others	Beyond control in everyday life
Provides a normality Physical and psychological improvements Brought a variety to everyday life Social connection Supports independence	Quality of life Faith in the future	Meaningfulness

All three researchers (KL, JG, ÅBT) were involved throughout the analytical process, and interpretations were discussed until consensus was reached. Reflexivity was maintained through ongoing discussions within the research team regarding pre-understandings and their potential influence on the analysis. The researchers were aware that previous involvement of KL and ÅBT in the intervention studies could influence interpretations toward expected benefits. These assumptions were actively discussed during analysis. Representative quotations were selected to illustrate the description categories and support transparency in the findings.

## 3 Results

Two description categories emerged from the analysis, “lifelong project leadership” and “meaningfulness”. These categories describe the outcome space of family and professional caregivers' perceptions of the long-term use of AEP after three to five years of use.

Family and professional caregivers perceived the long-term use of AEP for young people as a form of lifelong project leadership, involving careful planning of everyday life. In this planning, they had to balance aspects within their control facilitating exercise in the AEP, with aspects beyond their control hindering exercise in the AEP. When this balance was negatively affected, young peoplés opportunities for physical activity and exercise were disrupted. Achieving balance was essential for establishing structure and continuity in young people's physical activity and exercise. When such balance was attained, the caregivers perceived a meaningfulness associated with an improved quality of life for both the young people and for themselves, which gave them hope for the future.

### 3.1 Lifelong project leadership

Long-term use of AEP for the young people was perceived by the caregivers to be a lifelong project, in which everyday life relied on stability and was characterised by continuous responsibility for planning, coordination, and implementation over time. To make physical activity and exercise possible in young people's everyday life, caregivers needed to maintain control over multiple aspects of daily functioning. Establishing structure and balance required careful planning of everyday routines, through which physical activity and exercise were positioned as a central part of the young people's overall well-being.

Nevertheless, there were aspects that fell beyond the caregiveŕs control. Integrating physical activity and exercise into daily routines was perceived as a constant struggle, as they were dependent on external actors and societal resources, such as continuity of competent assistance and access to technical support for the AEP. Moreover, the caregivers expressed concerns, that over time it might be hard to sustain the existing level of physical activity and exercise, since they might not personally be around to support the young person.

#### 3.1.1 Maintaining control in everyday life

The caregivers' perception was that they had a lifelong leadership role; specifically, a project leadership role in which routines were essential for maintaining control and creating balance and structure in everyday life. This continuity provided them with a sense of security, and their overall well-being, as well as that of the young people in their charge, was positively affected.

The caregivers perceived physical activity and exercise as a central and important component of the young people's well-being, both physically and psychologically. However, they also understood that the positive effects of physical activity and exercise depended on everyday life being stable and on all involved parties contributing to a functional whole. They expressed a need to be able to trust that those involved, such as assistants, adhered to the structure they had planned.

“It has meant a lot, and it also happens on other levels. It's not always these weighing-and-measuring things, rather, it's another kind of satisfaction when you can say that something is happening. It's like it is among assistants, to get them to do something. Then it's good…now it's Saturday morning and you arrive at nine o’clock, and the first thing that happens is that now X is to get up and stand for an hour. It kind of fulfils several other functions in the context as well, so to speak. Because then everyone gets going, instead of it easily becoming a bit too laid-back. And he doesn't really like that.” (C01)

The responsibility for coordinating this entirety often rested on them. To meet the young people's needs, they had to plan, coordinate, and ensure continuity across different interventions/support measures. This included coordinating schedules, routines, and communication between assistants, healthcare personnel, and other services/activities the young people were involved in. As a result, there was very little room for spontaneity. When coordination failed, there was a risk that activities would be cancelled or reduced in quality, which could mean the young people missed out on important elements of daily stimulation. Maintaining control thus became a way for caregivers to protect the young people from disruptions in structure and to ensure continuity—both in physical activity and exercise, and among assistants.

“If you're going to get to activities, you have to have special transport (paratransit)…and you usually have to book it at least a day in advance. You can't really just come up with something on the spur of the moment, because then there's no transport. That's the kind of thing that also causes problems.” (C10)

Physical activity and exercise were perceived as an integral part of the young people's everyday life and needed to occur regularly. The caregivers perceived that the AEP facilitated this, as the device was available in the home. At the same time, the AEP was perceived as large and heavy and as taking up considerable space in the home. Furthermore, for the device to function in practice, young people required assistance to be placed into the AEP. This typically required one to two people, as well as additional equipment, such as a ceiling lift. Once the young people were in position, supervision was needed to notice whether the young people began to feel unwell or no longer wished to continue the activity.

“And I have said that as well, that you constantly have to look at how X is feeling…but he's actually quite good at saying so himself too, that he doesn't want to right now. Then he can sit down and tense up. And sort of not cry, but protest with sounds.” (C08)

The assistants therefore needed to be familiar with how the young people communicated and expressed emotions and needs. To encourage movement in the AEP, different forms of distraction were required. These were highly individual and could include watching TV, listening to music or stories, singing, dancing, or playing. The caregivers perceived that the AEP contributed to the young people's well-being on several levels, noting improvements in both psychological aspects, such as mood, and physical aspects, including enhanced bodily functions. At the same time, they expressed uncertainty about whether these improvements could be attributed solely to the AEP or whether multiple factors were at play.

#### 3.1.2 Beyond control in everyday life

Despite their everyday life being characterised by careful planning, caregivers perceived that there were aspects that fell beyond their control. It was a constant struggle to make everything work, and in this struggle, they found themselves in a position of dependence on other actors and on societal resources, circumstances they could not influence.

A central part of this struggle involved creating continuity among the assistants and ensuring that the young people were surrounded by people they could trust. All daily activities depended on how well this assistance was functioning. Without assistants, no physical activity and exercise could take place, and without physical activity and exercise, the sense of control, including control over the balance and structure of everyday life, collapsed. The caregivers also perceived that their position of dependence extended across many areas. The young people's safety was crucial, and they needed to be able to trust that the assistants not only had the appropriate training to operate the AEP, but also the competence to interpret and understand the young people; i.e., to read signals, recognise needs, and make the right decisions in the moment. The caregivers' perception was that the young peoplés well-being was tied to the presence, competence, and reliability of others. As long as the young people lived at home, the caregivers could influence these aspects, but they were aware that this control would diminish once the young people eventually moved into residential care.

“What I've talked a bit about is listening to the user, listening to X. Talking with her. There can be days when she's been very tired and you ask, “Are you going to get up and exercise?” No, then she looks toward the sofa instead. No. She doesn't want to, that day she doesn't have the energy. We don't always have the strength every day to go out and exercise. Most days she is very positive and shows that she wants to. But there are also days when you don't want to. Being attentive and reading the person who uses it, as well.” (C04)

“Not all staff have learned how it works. I think some staff find it difficult to do it. Mm. Yes, that some see it more as a problem than as a form of training for X.” (C03)

For the AEP to function in everyday life, they were dependent on technical support. As the AEP was used daily, it was crucial that the device operated as intended. Quick service and access to technicians were therefore necessary to avoid disruptions in the routines. Although they felt that the support from technicians generally worked well, the perception was that the responsibility to keep everything together still rested with them. They also expressed a need for more support from the habilitation services regarding the AEP, particularly with respect to training programs and routines. At present, they had to actively request support themselves, and they did not always receive the assistance they needed.

“I'm a bit worried about the lead times if this were handled through the assistive devices centre. It can take many weeks just to replace a screw there…speaking from experience. So that's what I would have been most…I have no complaints about the service or the way we're treated. I think that's excellent. But the lead times are terribly long. We live for long periods with unsatisfactory assistive devices, where we're just waiting either for an appointment or for a part. So that would definitely have been a concern.” (C02)

The caregivers expressed growing concern about the future as the young people grew older and became adults, particularly regarding support services, housing, and continued access to assistive devices. Physical activity and exercise were perceived as a human right, and they were afraid that the young people's opportunities for physical activity and exercise would decrease over time and fail to maintain the current standard. The AEP was perceived as an important tool to sustain mobility in everyday life.

“I'm worried that…that he won't get the amount of training and movement that he gets today, since it requires effort, as we've established. When it comes to his other activities, it's hard to see or believe that they will match what he has now. And then, of course, it would be very good, even better, to see it in context if you knew that you had an assistive device to fall back on that still gives him this movement while standing, so that he doesn't just end up sitting in his wheelchair in the future. Because it is, after all, an easier way to get him moving than the other methods.” (C07)

However, the right to have the AEP prescribed was not available in the region where they lived, which they found deeply unfair. To ensure that the young people still had access to the device, the families were forced to finance the purchase themselves, something that was not possible for everyone. Some applied for external funding to be able to buy the AEP, while others paid it out of pocket.

“I really think it's awful that it isn't equal, above all that it isn't the same across all of Sweden, at least that's what I've been told. In half of the country, you get it, and in the other half you don't. Just because we live in one part of Sweden, my daughter doesn't have the same opportunity as she would if she lived in another part.” (C04)

### 3.2 Meaningfulness

The perception was that physical activity and exercise provided meaningfulness, if the caregivers managed to balance everyday life. It was perceived as not only promoting young people's health, but also their quality of life by providing joy and opportunities for social participation. These positive effects were not limited to the young people alone but extended to the whole family, as the caregivers also experienced improved well-being, which increased their faith in the future.

“It means that I can offer my son real training every day, that means everything. Our other son we drive to his activities, and then he takes care of his training himself, but X can't do that. And the Innowalk means that X has a good alternative where he gets to train his whole body every day, that means a great deal to us. We also like to exercise and know what it gives you, the feeling you get, and, in general, everyone knows that physical activity is good for you.” (C02)

Physical activity and exercise in the AEP were perceived as contributing to an enhanced quality of life, both physically and psychologically. The caregivers described concrete physical effects, such as improved mobility and bowel function, better circulation as the feet became warmer, and easier breathing when mucus was loosened through the movement. Furthermore, they noticed an enhanced psychosocial well-being in the young people, as they often became more cheerful, more alert, and showed appreciation for the activity. The caregivers described that the young people generally expressed a sense that they wanted to use the AEP and that it felt pleasant to move in it. Even on occasions when the young peoplés motivation was low at first, the atmosphere usually changed once the young people were in place, as their mood improved and their energy levels increased.

“So, we clearly see a positive effect on her stomach and bowel function. We do. Then we sometimes wonder whether it might also be good for the airways and lung capacity. That's harder to know, but we do think it probably is. Because she is generally quite healthy these days. When she was little, she was ill much more often. That can have many different causes. We suspect that being active and moving may also have a positive effect on that. It feels logical, at least.” (C09)

“If you really push it, we don't actually know how much real effect we can see from anything. But what has been the case is that X has always been extremely comfortable as soon as he got up into the Innowalk. We've always experienced that once he gets going, he becomes alert. He is usually very happy when he's standing there and when he's busy doing things. He never becomes passive, so to speak. That has been a great benefit for us.” (C01)

The caregivers' perception was that the AEP gave the young people a sense of normality. The AEP enabled the young people to come into a standing position, something that is taken for granted in everyday life for most people. In a standing position, the young people had the opportunity to move in a way that resembled how others move and were able to move independently. Additionally, the AEP brought variety to an otherwise monotonous everyday life. It also provided the young people an opportunity to maintain mobility and stretch the body in ways that might otherwise be difficult to achieve.

“That's good for everyone, not always being in the same position. But yes, it creates a change of position, she becomes stronger in her body by stretching out. She doesn't become, I don't know what it's called, but she doesn't get stiff in the same way if she's able to get up and move like that.” (C03)

“We believe that it benefits her. She usually enjoys being in it. That's why it feels meaningful and good for us to have it as something to do with her, or to let her do during the day. If you're at home for a whole day, for example, on the weekend, it's good to have a few different activities to do so that the day feels meaningful and worthwhile. Otherwise, it can become a bit monotonous. It becomes something enjoyable for her. It also feels more natural for the rest of us, in a way, you're more active at times, and then there's more relaxation afterward. It makes for a better everyday life.” (C09)

The caregivers also perceived that when the young people were in the AEP, it offered an alternative form of social connection. The situation became free of demands; they could talk, laugh, and be together without being weighed down by the practical ‘musts’ that otherwise characterised their everyday life. These moments in the AEP were therefore perceived as meaningful not only for the young people, but also for the family, which strengthened their optimism about the future.

“For me, the fact that X can use this machine (the Innowalk) has meant that I, as a father, feel better myself. What I mean is that I know she has a good device that really helps her. And in that way, I feel much better.” (C5)

“Basically, if X is doing well, then I'm doing well too.” (C04)

## 4 Discussion

In the interviews about physical activity and exercise in the AEP with the caregivers, the caregivers talked about being a balancing act between maintain control and factors beyond control in daily life, to provide physical activity and exercise in the AEP. The balancing act was described as a form of lifelong project leadership, entailing coordination of support from personal assistants and handling governmental decisions, and being dependent on others. When the caregiver managed to balance all this, and were able to provide physical activity and exercise through the AEP, using the AEP was perceived as rewarding and as creating a feeling of meaningfulness.

In an earlier study about caregiver's experience of the AEP when it was first introduced (15), the caregivers experienced that the AEP gave them hope for the future of their child. After three to five years of using the AEP, the hope has changed into meaningfulness after seeing how their child is enjoying and thriving in the AEP. Furthermore, caregivers highlighted that the AEP not only promoted physical activity but also enabled the young person to experience a sense of normality by attaining an upright standing position and moving independently of others. When first introduced to the AEP, the caregivers experienced being dependent on others for access to an accessible environment and the AEP (15). The caregivers still spoke about dependence and how this is part of their lifelong project leadership. Others have described that being a caregiver to a child with CP-NA often involves a lifelong responsibility characterised by extensive caregiving, coordination of healthcare services, and continuous adaptation to the child's individual needs. Consequently, children with CP-NA are frequently dependent on their caregivers in daily activities as well as in interactions with healthcare providers, rehabilitation services, and social support systems (19). Dependency on support for children with CP-NA to be physically active has also been recognised among healthcare professionals (20). This is similar to what we have found when caregivers speak about how they have to manage habilitation services, technical aid services, and the support system offering personal assistance to provide physical activity and exercise in the AEP.

Additionally, research demonstrates that caregivers of children with CP-NA often describe caregiving as an ongoing and demanding process that affects the entire family's life. In a meta-synthesis of qualitative studies, Dlamini et al. described how parents gradually assume the role of coordinators and leaders of their child's care and rehabilitation. Caregivers are responsible for medical appointments, rehabilitation, financial management, planning, and daily caregiving, making caregiving a lifelong project rather than a temporary task (21). This experience can be understood as a form of ‘lifelong project leadership’, in which caregivers continuously organise and sustain the child's living conditions and well-being. Several studies also describe how caregivers strive to create control and structure in an everyday life often characterised by uncertainty and unpredictability. Caregivers develop routines, expert knowledge, and coping strategies to manage their child's needs and to create stability within family life (22). Within this form of lifelong project leadership, the structure the caregivers establish functions as a protective framework that supports the child's safety, well-being, and sense of being loved (23). At the same time, many caregivers experience limited ability to influence the long-term consequences and progression of the child's condition. In our study, caregivers expressed concern about the transition to independent living, particularly regarding access to support services and suitable housing. Furthermore, they were also concerned about a potential decline in physical activity, given the positive benefits they had observed. This experience of facing situations beyond one's control is associated with physical and emotional exhaustion, financial strain, social isolation, and concerns about the future (24). Furthermore, research also shows that caregiving significantly affects parents' psychological and social well-being. Mothers of children with CP frequently report higher levels of stress, fatigue, and depressive symptoms compared to parents of children without disabilities (25). Qualitative studies describe how continuous caregiving responsibility impacts employment, relationships, and opportunities for recovery and rest. Parents also express feelings of loneliness and insufficient support from healthcare systems and society (24).

Despite these challenges, experiences of meaning and personal growth are also evident in the literature. Caregivers describe how their relationship with the child can foster a profound sense of love, responsibility, and existential meaningfulness. Phenomenological studies indicate that many parents develop new perspectives on life and discover personal strength through the caregiving role, despite the difficulties they encounter in everyday life (24). Meaning-making therefore appears to coexist with experiences of burden and loss of control.

Overall, previous research suggests that caregivers of children with CP live in a complex life situation in which responsibility, control, uncertainty, and meaningfulness constitute central dimensions of their lived experience. Although several studies have explored caregiver burden and psychosocial impact, there remains a need for deeper understanding of how parents experience their long-term caregiving responsibility from a lifeworld perspective, particularly in relation to issues of control, responsibility, and meaning (21).

### 4.1 Strengths and limitations

In phenomenographic studies, pre-understanding is important, meaning that the researchers need to be aware of the phenomenon and how it may be perceived (16). The research team represented diverse disciplinary backgrounds and areas of expertise, including two physiotherapists with extensive knowledge of the field and one radiographer with expertise in qualitative research but without prior familiarity with the specific field. This interdisciplinary composition contributed to enhanced credibility by enabling multiple perspectives during data interpretation and reducing the potential risk of bias while maintaining methodological rigor.

As the study was a follow up of long-term use (3–5 years) of the AEP, and prior contact with the participants had already been established. This prolonged and careful engagement contributed to building trust and facilitated rich and nuanced data, despite the interviews being conducted at the participants' home, online, or by telephone. However, the existing relationship between participants and members of the research team may also have influenced how perceptions were described and interpreted. Additionally, the circumstance that only one professional caregiver was included among the informants could be both a limitation and a to be considered a strength of the study, since the professional caregiver has another perspective from the family caregivers. Moreover, families with negative experiences may have discontinued use of the AEP and therefore been less likely to participate in the follow-up interviews. Consequently, the findings may overrepresent caregivers with more positive experiences of long-term use. To support transferability, a detailed description of the context and participants' characteristics were provided.

To enhance the dependability of the analysis, three researchers (KL, JG, ÅBT), all of whom are experienced in qualitative interview analysis, were involved throughout the analytical process. Several meetings were conducted to discuss similarities and differences, and interpretations were refined until consensus was reached. Confirmability was strengthened through the use of quotations from the participants to illustrate and support the findings.

## 5 Conclusion

These findings suggest that the long-term use of an AEP can be both challenging and rewarding to caregivers to young people with CP-NA. With the right support, the use of an AEP can give the caregivers a sense of meaningfulness in a challenging situation. Long-term use of the AEP is not solely dependent on the device itself but on a wider support system, including personal assistance, technical services, and rehabilitation professionals. Supporting caregivers in coordinating these resources may be essential for sustaining physical activity over time.

For caregivers of young people with CP-NA, physical activity and exercise represented more than a health-promoting intervention, it was perceived as a cornerstone of everyday life and well-being. The AEP was viewed as an important facilitator and, despite the challenges associated with its long-term use, contributed to a sense of meaningfulness and hope for the future. However, successful use of the AEP was not dependent on the AEP alone but on a fragile interplay between family efforts, competent personal assistance, healthcare services, technical support, and societal resources. Caregivers assumed a lifelong leadership role in coordinating these resources while simultaneously expressing concerns about the sustainability of current arrangements as the young people transitioned into adulthood. Addressing organisational barriers and inequities in access to AEP, together with strengthening support for caregivers, may be essential for upholding the right to physical activity and promoting health, well-being, and participation throughout adulthood.

## Data Availability

The datasets presented in this article are not readily available because the data used in this study includes sensitive personal information, and the participants did not consent to public sharing of their data. The current ethical approval from the Regional Ethical Review Board in Lund, Sweden (2019–00106) does not include data sharing. A limited dataset may be made available upon request to a qualified academic investigator for the sole purpose of replicating the present study, provided the data transfer is in agreement with EU legislation on the general data protection regulation and approval by the Swedish Ethical Review Authority. Requests to access the datasets should be directed to Åsa B Tornberg, asa.tornberg@med.lu.se.
