# Supplementary material for: Long-term use of an assistive exercise product in young people with cerebral palsy who are non-ambulant is a balancing act—a phenomenographic study
Source: Front Rehabil Sci. 2026 Sep 23;7:1927259. doi: 10.3389/fresc.2026.1927259 (PMC13642274; doi:10.3389/fresc.2026.1927259)
Supplement: Supplementary file 1 [file Datasheet1.pdf]

Supplementary Material:
Supplement 1.

COREQ (Consolidated criteria for reporting qualitative research) Checklist

A checklist of items that should be included in reports of qualitative research. You must report the page number in your manuscript where you consider each of the items listed in this checklist. If you have not included this information, either revise your manuscript accordingly before submitting or note N/A.

Topic	Item No.	Guide Questions/Description	Reported on Page No.
**Domain 1: Research team and reflexivity**			
Personal characteristics			
Interviewer/facilitator	1	Which author/s conducted the interview or focus group?	5
Credentials	2	What were the researcher's credentials? E.g. PhD, MD	Front page
Occupation	3	What was their occupation at the time of the study?	12
Gender	4	Was the researcher male or female?	N/A
Experience and training	5	What experience or training did the researcher have?	12
Relationship with participants			
Relationship established	6	Was a relationship established prior to study commencement?	4
Participant knowledge of the interviewer	7	What did the participants know about the researcher? e.g. personal goals, reasons for doing the research	3,4
Interviewer characteristics	8	What characteristics were reported about the inter viewer/facilitator? e.g. Bias, assumptions, reasons and interests in the research topic	4,12
**Domain 2: Study design**			
Theoretical framework			
Methodological orientation and Theory	9	What methodological orientation was stated to underpin the study? e.g. grounded theory, discourse analysis, ethnography, phenomenology, content analysis	4
Participant selection			
Sampling	10	How were participants selected? e.g. purposive, convenience, consecutive, snowball	4
Method of approach	11	How were participants approached? e.g. face-to-face, telephone, mail, email	4
Sample size	12	How many participants were in the study?	4
Non-participation	13	How many people refused to participate or dropped out? Reasons?	4
Setting			
Setting of data collection	14	Where was the data collected? e.g. home, clinic, workplace	4
Presence of non-participants	15	Was anyone else present besides the participants and researchers?	5
Description of sample	16	What are the important characteristics of the sample? e.g. demographic data, date	4, table 2
Data collection			
Interview guide	17	Were questions, prompts, guides provided by the authors? Was it pilot	Supplement 2,

		tested?	5
Repeat interviews	18	Were repeat inter views carried out? If yes, how many?	N/A
Audio/visual recording	19	Did the research use audio or visual recording to collect the data?	5
Field notes	20	Were field notes made during and/or after the interview or focus group?	5
Duration	21	What was the duration of the inter views or focus group?	5
Data saturation	22	Was data saturation discussed?	5
Transcripts returned	23	Were transcripts returned to participants for comment and/or	N/A

Topic	Item No.	Guide Questions/Description	Reported on Page No.
		correction?	
**Domain 3: analysis and findings**			
Data analysis			
Number of data coders	24	How many data coders coded the data?	NA
Description of the coding tree	25	Did authors provide a description of the coding tree?	5, table 2
Derivation of themes	26	Were themes identified in advance or derived from the data?	5, table 2
Software	27	What software, if applicable, was used to manage the data?	N/A
Participant checking	28	Did participants provide feedback on the findings?	N/a
Reporting			
Quotations presented	29	Were participant quotations presented to illustrate the themes/findings? Was each quotation identified? e.g. participant number	6-10
Data and findings consistent	30	Was there consistency between the data presented and the findings?	6-10
Clarity of major themes	31	Were major themes clearly presented in the findings?	6-10
Clarity of minor themes	32	Is there a description of diverse cases or discussion of minor themes?	6-10

Developed from: Tong A, Sainsbury P, Craig J. Consolidated criteria for reporting qualitative research (COREQ): a 32-item checklist for interviews and focus groups. *International Journal for Quality in Health Care*. 2007. Volume 19, Number 6: pp. 349 – 357

Supplement 2. Interview guides pre and post (original Swedish)

Intervjuguide: Före patientutbildningen

1. Kan du berätta lite om dig själv?
2. Bakgrund, erfarenheter (arbete och andra), familj mm
3. Kan du berätta lite om din sjukdom och hur den påverkar din vardag?
4. Vilka symtom upplever du som mest besvärande?

Kunskap och uppfattningar om sjukdomen

5. Hur hanterar du din medicinering och symtom i dagsläget?
6. Vilka utmaningar upplever du med att följa din behandling?
7. Hur skulle du beskriva din kunskap om Parkinsons sjukdom och dess behandling?
8. Har du tidigare deltagit i någon utbildning eller informationsträff om Parkinsons sjukdom?

Förväntningar på utbildningen

9. Vad hoppas du få ut av den här patientutbildningen?
10. Finns det något specifikt du vill lära dig mer om?
11. Hur tror du att utbildningen kan påverka din vardag och din sjukdomshantering?

Avslutning

12. Vill du lägga till något?

Intervjuguide: Efter patientutbildningen

1. Berätta för mig om patientutbildningen
2. Hur upplevde du utbildningen?
3. Var den som du hade förväntat dig?
4. Vilka delar av utbildningen tyckte du var <u>mest användbara</u>?
5. Fanns det något du saknade eller som kunde ha <u>förbättrats</u>?
6. Hur har det funkat med formatet? E.g. digitala möten

Förändringar i kunskap och hantering av sjukdomen

7. Hur har din förståelse för Parkinsons sjukdom förändrats efter utbildningen?
    a. På vilket sätt?
8. Har du ändrat hur du hanterar din medicinering och symtom?
    a. Om ja, hur?
9. Upplever du att utbildningen har påverkat din livskvalitet?

Framtida behov och stöd

10. Finns det något du fortfarande känner att du behöver mer information eller stöd kring?
11. Hur tror du att vården kan stödja dig bättre i din sjukdomshantering?
12. Skulle du rekommendera den här typen av utbildning till andra med Parkinsons sjukdom?
    a. Varför/varför inte?

Avslutning:

13. Vill du lägga till något?

Supplement 2. Interview guides pre and post (translated to English)

Interview guide: Before the LC

1. Tell me a little about yourself.
2. Background, experiences (work and others), family, etc.
3. Can you tell me about your PD and how it affects your everyday life?
4. Which symptoms do you find most bothersome?

Knowledge and perceptions about the illness

5. How do you currently manage your medication and symptoms?
6. What challenges do you experience in following your treatment (if any)?
7. How would you describe your knowledge of Parkinson's disease and its treatment?
8. Have you previously participated in any training or information meeting about Parkinson's disease?

Expectations for the training

9. What do you hope to get out of this LC?
10. Is there anything specific you would like to learn more about?
11. How do you think the training could affect your everyday life and your disease management?

In closing

12. Is there anything you would like to add?

Interview Guide: After the LC

1. Tell me about the LC.
2. How did you experience the LC?
3. Was it what you expected?
4. Which parts of the LC did you find most useful?
5. Was there anything you missed or that could have been improved?
6. How did the format work? E.g. digital meetings

Changes in knowledge and management of the disease

7. How has your understanding of Parkinson's disease changed after the LC?
    a. In what way?
8. Have you changed how you manage your medication and symptoms?
    a. If yes, how?
9. Do you feel that the education has affected your quality of life?

Future needs and support

10. Is there anything you still feel you need more information or support on?
11. How do you think healthcare can support you better in managing your disease?
12. Would you recommend this type of education to others with Parkinson's disease?
    a. Why/why not?

In closing:

13. Is there anything else you would like to add?
